# Chidamide Suppresses the Growth of Cholangiocarcinoma by Inhibiting HDAC3 and Promoting FOXO1 Acetylation

**DOI:** 10.1155/2022/3632549

**Published:** 2022-01-28

**Authors:** Yongpan Li, Jujia Zheng, Qiang Huo, Zhongchao Chen, Jun Chen, Xiangwei Xu

**Affiliations:** ^1^Department of Hepatopancreatobiliary Surgery, The First People's Hospital of Yongkang, Affiliated to Hangzhou Medical College, Yongkang 321300, China; ^2^School of Pharmaceutical Sciences, Wenzhou Medical University, Wenzhou 325035, China; ^3^Department of Hepatopancreatobiliary Surgery, Putuo Hospital of Zhejiang Province, Zhoushan 316100, China; ^4^Department of Pharmacy, The First People's Hospital of Yongkang, Affiliated to Hangzhou Medical College, Yongkang 321300, China

## Abstract

Inhibitors for histone deacetylases (HDACs) have been identified as epigenetic drug targets to treat a variety of malignancies through several molecular mechanisms. The present study is aimed at investigating the mechanism underlying the possible antitumor effect of the HDAC inhibitor chidamide (CDM) on cholangiocarcinoma (CCA). Microarray-based gene expression profiling was conducted to predict the expression of HDACs in CCA, which was validated in clinical tissue samples from CCA patients. Next, the proliferation, migration, invasion, autophagy, and apoptosis of human CCA QBC939 and SNU308 cells were measured following treatment with CDM at different concentrations. The acetylation level of FOXO1 in the nucleus and cytoplasm of QBC939 and SNU308 cells was determined after overexpression and suppression of HDAC3. A QBC939-implanted xenograft nude mouse model was established for further exploration of CDM roles *in vitro*. HDAC3 was prominently expressed in CCA tissues and indicated a poor prognosis for patients with CCA. CDM significantly inhibited cell proliferation, migration, and invasion of QBC939 and SNU308 cells, while inducing their autophagy and apoptosis by reducing the expression of HDAC3. CDM promoted FOXO1 acetylation by inhibiting HDAC3, thereby inducing cell autophagy. Additionally, CDM inhibited tumor growth *in vivo via* HDAC3 downregulation and FOXO1 acetylation induction. Overall, this study reveals that CDM can exhibit antitumor effects against CCA by promoting HDAC3-mediated FOXO1 acetylation, thus identifying a new therapeutic avenue for the treatment of CCA.

## 1. Introduction

Cholangiocarcinoma (CCA) is a common biliary malignancy with increased incidence rate in association with suspected risk factors such as obesity and hepatitis C virus infection [1, 2]. Due to the present absence of sensitive early biomarkers, diagnosis of CCA is often delayed to an advanced stage of this disease, such that five-year survival is less than 10% [3]. The available treatments include targeted, combined chemotherapy and immunotherapy and personalized therapies, but there is not curative treatment for advanced disease.

Chidamide (CDM), a novel histone deacetylase (HDAC) inhibitor, has been reported to play an antitumor role in T cell tumors through multiple mechanisms [4]. In multiple myeloma cells, CDM induces growth arrest and apoptosis in a caspase-dependent manner [5], but CDM is scantly researched in the context of CCA. Histone deacetylases (HDACs) are a family of enzymes capable of catalyzing the removal of acetyl groups from the acetyl-lysine residues in histone and nonhistone proteins [6]. HDACs exert a vital function in numerous cancers through their regulation of the expression and function of proteins involved in cancer initiation and progression [7]. Selective inhibitors for HDACs have been identified as treatments for epigenetic targets to treat a variety of malignancies through several molecular mechanisms [8, 9]. HDAC inhibitors have also demonstrated potent and specific anticancer stem cell activities; CDM can specifically promote apoptosis of leukemia stem cell-like cells and primary acute myeloid leukemia CD34(+) cells in a concentration- and time-dependent manner [10]. In addition, HDAC2 is an essential factor regulating cancer stem cell phenotype as its silencing can promote the stemness of MG63 and Saos2 cells [11]. HDAC inhibitors can inhibit the migration and invasion of CCA cell lines [12], and other research shows that the HDAC inhibitor CG200745 exerts antitumor effects in CCA cell lines via miRNAs targeting the Hippo pathway [13]. HDAC inhibitors can act on the cellular stress response pathway, reduce angiogenesis through downregulation of angiogenic genes such as VEGF, HIF-1, and eNOS to inhibit the formation of new blood vessels and, in combination with classic chemotherapy drugs, interfere with CCA cell migration [14]. Published research has reported that the HDAC inhibitor CDM synergizes with Rituximab to inhibit the growth of diffuse lager B-cell lymphoma tumors by regulating CD20 [15]. As one of the potential targets of CCA, FOXO1 bears a close relationship to the sensitivity of tumors to drugs [16]. In CCA cells, the interaction between FOXO1 acetylation and Atg7 regulates autophagic flux, promotes cell apoptosis, and exerts an antitumor effect [17]. Metformin enhances autophagy *via* the AMPK/SIRT1-FOXO1 pathway in diabetic kidney disease, which suggests the involvement of FOXO1 in autophagy [18]. By promoting SIRT1-FOXO1-ATG14 axis-dependent autophagy, paeonol prevents palmitic acid-induced dysfunction of lipid metabolism in HepG2 injury [19]. However, there are hitherto no reports of CDM in CCA. In this study, wet set out to explore whether CDM can play an antitumor effect in CCA cells and to test the mechanism associated with HDAC3-mediated deacetylation of FOXO1.

## 2. Materials and Methods

### 2.1. Ethics Statement

The current study was approved by the Ethics Committee of the First People's Hospital of Yongkang, Affiliated to Hangzhou Medical College, and performed in strict accordance with the *Declaration of Helsinki*. All participants signed informed consent documentation before sample collection. The animal experiments were performed with the approval from the experimental animal institution.

### 2.2. Cell Screening, Culture, and Transfection

Human CCA cell lines QBC939 and SNU308 purchased from Shunran Biological Technology Co., Ltd. (Shanghai, China) were cultured in Dulbecco's modified Eagle's medium (DMEM) (Gibco Invitrogen Co., USA) with 10% fetal bovine serum (Gibco), 10 *μ*g/mL streptomycin, and 100 *μ*g/mL penicillin in a 5% CO_2_ incubator at 37°C. Cells in the logarithmic growth phase were collected after trypsin digestion and seeded in 6-well plates at a density of 1 × 10^5^ per well and cultured normally for 24 hours. Upon reaching about 75% confluence, the cells were transfected according to the instructions of Lipofectamine 2000 reagent (Invitrogen) with sh-NC (5′-CACCGCTTCCATTCTGAGGACTACACGAA-3′), sh-HDAC3 (5′-AAAAGCTTCCATTCTGAGGACTACATTCG-3′), oe-NC (forward: CCGCTCGAGATGAGGCCGCGGCAT, reverse primer: CCGCAATTCGAGTCACCFCCTCCTGT), and oe-HDAC3 plasmids (forward: CCGCTCGAATTCTGAGGAGCAT, reverse: CCGCAATTCGAGGCCGFCCTCCTGT). After transfection for 48 hours, the transfection efficiency of sh-HDAC3 and oe-HDAC3 was measured by Western blotting. The plasmids were purchased from Shanghai GenePharma Co. Ltd. (Shanghai, China) and used at a concentration of 50 ng/mL, whereas the CDM (C860936, Seedior, Shanghai, China) treatments were 2.5 *μ*M (CDM low, CDM-L), 5 *μ*M (CDM middle, CDM-M), and 10 *μ*M (CDM high, CDM-H).

### 2.3. Cell Counting Kit-8 (CCK-8) Assay

QBC939 and SNU308 cells were maintained in DMEM complete medium supplemented with 10% FBS at 37°C in a 5% CO_2_ incubator. QBC939 and SNU308 cells were seeded onto 96-well plates at a density of 1 × 10^4^ cells/well, with 90 *μ*L cell suspension per well, whereas the blank control was incubated in 90 *μ*L medium. After culture overnight, the cells were treated with CDM of different concentrations to a final concentration of 0.1, 0.5, 1.0, 2.5, 5.0, 10.0, and 20.0 *μ*M. The control and the blank control wells were incubated only with 10 *μ*L medium. There were six replicate wells for each treatment. After 48 hours, 10 *μ*L CCK-8 test solution was added to each well and then incubated for 1 hour, whereupon the absorbance was measured at 450 nm using a microplate reader. Cell inhibition rate was calculated through the formula which is cell inhibition rate = (*A*_control_–*A*_CDM_)/(*A*_control_–*A*_blank control_) × 100%. The experiment was repeated three times independently.

### 2.4. Scratch Test

Logarithmic growth phase BC939 and SNU308 cells were evenly inoculated into 2 wells (70 *μ*L/well) at a density of 4 × 10^5^ cells/well in the scratch insert placed in a small Petri dish. A small portion of serum was added to the Petri dish and incubated in the incubator overnight. The next day, when the cells had adhered stably, the scratch plug was carefully removed. The cells were washed gently twice with PBS, 900 *μ*L of serum-free medium was added, and then the cells were photographed immediately (0 hour). Subsequently, 100 *μ*L CDM at concentrations of 25, 50, and 100 *μ*M was added to experimental groups, and 100 *μ*L medium was added to the control dishes. After culture in the incubator for 48 hours, the cells were photographed. The distance between the two holes of the scratch insert was 0.5 mm. The scratch difference value of two photographs was the migration distance of CCA cells. The experiment was repeated three times independently.

### 2.5. Colony Formation Assay

Following treatment with CDM for 48 hours, QBC939 and SNU308 cells were trypsinized, seeded into a 6-well plate (approximately 600 cells per well), and cultured for 14 days to form clonal colonies. Next, the cells were fixed with formaldehyde (Shanghai Pudong Jizhihua Co., Ltd., Shanghai, China), stained with 0.1% crystal violet (Baoman Biotechnology, Yangpu, Shanghai, China), and counted under a microscope. The experiment was repeated three times independently.

### 2.6. Flow Cytometry

Following treatment with CDM for 48 hours, QBC939 and SNU308 cells were collected and washed three times with PBS. The cells were suspended in 195 *μ*L Annexin V Binding Buffer in the dark and stained with 5 *μ*L Annexin V-FITC and 10 *μ*L PI within five minutes, for a total of 20 minutes. Subsequently, the samples were detected by FACS flow cytometry and analyzed by FlowJ. The experiment was repeated three times independently.

### 2.7. Transwell Assay

The EC Matrigel thawed at 4°C overnight was diluted 1 : 9 with serum-free medium to a final concentration of 1 mg/mL, whereupon 40 *μ*L of the mixture was added to the polycarbonate membrane of a 24-well precooled transwell chamber. Then, the chambers were incubated for 5 hours in an incubator containing 5% CO_2_ at 37°C to polymerize the EC Matrigel to form a gel. After discarding the excess liquid, pure DMEM salutation at a dose of 70 *μ*L/chamber was added and incubated at 37°C for 0.5 hours to rehydrate the matrix glue and remove the excess medium for next use. After removing the serum for 24 hours, the cells were digested, centrifuged, and resuspended in DMEM without FBS to a final concentration of 2.5 × 10^5^ cells/mL. Next, 200 *μ*L of cell suspension was added into the upper chamber where the basement membrane had been hydrated, and 700 *μ*L of pre-cooled DMEM containing 10% FBS was added to the lower chamber. After incubation in a 37°C, a 5% CO_2_ humidity incubator for 24 hours, the chamber was removed and the cells in the chamber and basement membrane were wiped off with a cotton bud. The chamber was fixed with methanol for 30 minutes and then stained with 0.1% crystal violet for 20 minutes. Finally, the chamber was inverted to air-dry, observed under an inverted microscope, and photographed. We counted the number of cells passing through the membrane in five randomly selected visual fields and averaged.

### 2.8. Western Blotting

Cells were collected after trypsin digestion and lysed with enhanced RIPA buffer containing protease inhibitor (Boshide Co., Ltd., Wuhan, China), and protein concentration was determined by a BCA Protein Assay Kit (Boshide Co., Ltd., Wuhan, China). Equal amounts of protein were separated by 10% SDS-PAGE and transferred to a PVDF membrane, followed by blockade of nonspecific binding with 5% BSA at room temperature for 2 hours. Subsequently, membranes were incubated at 4°C overnight with primary rabbit antibodies to HDAC3 (Ab32369, 1 : 2000, Abcam), LC3B (ab51520, 1 : 2000, Abcam), ULK1 (ab203207, 1 : 2000, Abcam), Beclin-1 (ab207612, 1 : 1000, Abcam), and Ac-FOXO1 (AF2305, 1 : 1000; Affinity Biosciences, Jiangsu, China). The membranes were the incubated with HRP-conjugated goat anti-rabbit secondary antibody (ab205719, 1 : 2000, Abcam) at room temperature for 1 hour. ECL working solution (EMD Millipore, USA) was applied for 1 minute at room temperature to visualize membranes. ImageJ was used to quantify the gray levels of each band, with GADPH (rabbit, ab181602, 1 : 10000) as the loading control. The experiment was repeated three times.

### 2.9. Clinical Samples

Cancer tissues were collected from 26 patients (16 males and 10 females, aged 47-73 years, with mean age of 60.1 ± 8.4 years) with CCA undergoing surgical resection at the First People's Hospital of Yongkang, Affiliated to Hangzhou Medical College. CCA was confirmed by postoperative pathological section. Patients with distant metastases and cachexia were excluded from the study. CCA tissues were obtained following surgery and then fixed with 70% ethanol and sectioned to observe histological characteristics. During the dissection process, the affected area was carefully outlined. Moreover, areas infiltrated by immune cells and blood vessels were excluded from the samples to minimize contamination. In addition, the normal bile duct tissues of 26 patients who underwent pancreaticoduodenectomy at the First People's Hospital of Yongkang, Affiliated to Hangzhou Medical College, were collected as the control [20].

### 2.10. Immunohistochemistry

Paraffin sections of clinical tissue samples were dewaxed, rehydrated, and treated with 3% methanol-H_2_O_2_ for 20 minutes. Next, the sections were washed with 0.1 M PBS for 3 min, followed by antigen retrieval in a water bath. The sections were blocked with normal goat serum (C-0005, Shanghai Haoran Biological Technology Co., Ltd., Shanghai, China) at room temperature for 20 minutes and immunostained with primary antibody to HDAC3 (Ab32369, 1 : 2000; Abcam) and Ac-FOXO1 (AF2305, 1 : 200, affinity Biosciences) overnight at 4°C. The next day, the sections were incubated with secondary antibody goat anti-rabbit IgG (ab6785, 1 : 1000, Abcam) at 37°C for 20 minutes. The HRP-labeled streptavidin protein working solution (0343-10000 U, Yimo Biotechnology Co., Ltd., Beijing, China) was added to the sections for incubation at 37°C for 20 minutes. DAB (ST033, Guangzhou Weijia Technology Co., Ltd., Guangzhou, China) was used to develop the sections, which were counterstained with hematoxylin (PT001, Shanghai Bogu Biotechnology Co., Ltd., China, Shanghai) for 1 minute. The sections were blued in 1% ammonia water, dehydrated, and cleared and mounted before observation under a microscope in 5 randomly selected high-power fields from each section, with 100 cells counted in each field. The experiment was repeated three times.

### 2.11. Immunoprecipitation (IP)

Cells were lysed in lysis buffer containing 50 mM Tris-HCl (pH 7.4), 150 mM NaCl, 10% glycerol, 1 mM EDTA, 0.5% NP-40, and protease inhibitor mixture, and the cell debris was eliminated by centrifugation. The cell lysate was then incubated with 1 *μ*g anti-FLAG antibody (ab125243, Abcam) and 15 *μ*L protein A/G beads (Santa Cruz Biotechnology) for 2 hours. After extensive washing, the beads were placed in a boiling water bath for 5 minutes. The proteins were separated by SDS-PAGE, transferred to nitrocellulose membrane (Millipore, Temecula, CA, USA), and then subjected to Western blotting to evaluate the acetylation level of FOXO1.

### 2.12. Xenograft Tumor in Nude Mice

Thirty-six healthy nude mice (Beijing Institute of Pharmacology, Chinese Academy of Medical Sciences, Beijing, China) aged 6-8 weeks were housed individually in an SPF animal laboratory with humidity 60–65% and temperature 22–25°C at a 12 h light/dark cycle, with free access to food and water. Experiments were conducted after one week of acclimatization.

Using lentivirus LV5 (GenePharma), QBC939 cells stably transfected with HDAC3 overexpression or overexpression control were constructed. The cells (100 *μ*L, about 1.0 × 10^7^ cells) were then injected subcutaneously into the left shoulder of the mice. The mice were grouped into Ctrl, CDM-H (intraperitoneal injection of 90 mg/kg CDM), CDM-M (intraperitoneal injection of 45 mg/kg CDM), CDM-L (intraperitoneal injection of 22.5 mg/kg CDM), CDM-H+oe-HDAC3 (intraperitoneal injection of 90 mg/kg CDM and lentivirus carrying oe-HDAC3), and oe-NC (injection of lentivirus carrying oe-NC), with 6 mice in each group. Administration was conducted twice a week for a total of 2 weeks. Tumor volume was evaluated every 5 days and calculated using the formula: volume = 0.5 × length × width^2^. After 20 days, the mice were euthanized, after which all tumors were excised, weighed, and photographed. In addition, the heart, liver, spleen, lung, and kidney of mice in each group were collected, and the distribution ratio of CDM in tumor tissues and each organ was measured with UV at 258 nm.

### 2.13. H&E Staining

The tumor tissue samples were rinsed with normal saline, fixed in 4% paraformaldehyde for 30-50 minutes, dehydrated, dewaxed, embedded in paraffin, and sectioned. The tissue sections were flattened and pasted on a glass slide, dried in a 45°C calorstat, dewaxed, and rehydrated. After rinsing with distilled water for 5 minutes, the sections were stained with hematoxylin for 5 minutes, washed in running water for 3 seconds, differentiated in 1% hydrochloric-acid ethanol for 3 seconds, and counterstained with 5% eosin solution for 3 minutes. Thereafter, the sections were dehydrated, cleared, and sealed. Finally, the tissue sections were visualized under a fluorescence microscope.

### 2.14. Microarray-Based Gene Expression Profiling

UALCAN database (http://ualcan.path.uab.edu/) was applied to determine the expression of HDAC I (HDAC1, 2, 3, and 8), II (HDAC4, 5, 6, 7, 9, and 10), and IV (HDAC11) in CCA based on TCGA database and the Kaplan-Meier survival curve of gene expression in patients with CCA.

### 2.15. Statistical Analysis

All measurement values were presented as the mean ± standard deviation and analyzed by SPSS 21.0 (IBM Corp. Armonk, NY, USA). An unpaired *t-*test was employed for data comparison between two groups, while one-way ANOVA or repeated measures ANOVA with Tukey's post hoc test was used for multigroup data comparison. *p* < 0.05 was considered statistically significant.

## 3. Results

### 3.1. HDAC3 Is Highly Expressed in CCA Tissues and Is Associated with Poor Prognosis of Patients

**Figure 1:**
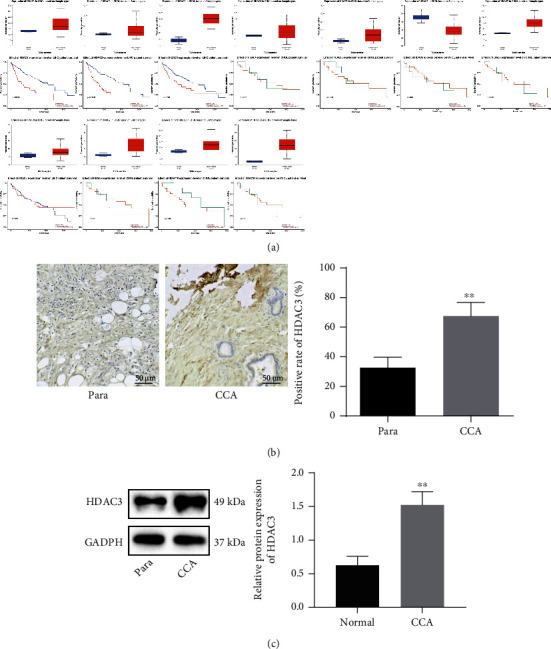
Amplified HDAC3 in CCA tissues indicates a poor prognosis of CCA patients. (a) Expression of HDAC I (HDAC1, 2, 3, and 8), II (HDAC4, 5, 6, 7, 9, and 10), and IV (HDAC11) in normal (*n* = 9) and CCA samples (*n* = 36) in TCGA database, as well as the correlation between HDACs and the survival rate of patients with CCA. (b) Immunohistochemistry of HDAC3 protein in cancer tissues (*n* = 26) and normal bile duct tissues (*n* = 26). (c) Western blotting of HDAC3 protein in cancer tissues (*n* = 26) and normal bile duct tissues (*n* = 26). ^∗^*p* < 0.05, compared to the normal bile duct tissues. Data were shown as the mean ± standard deviation. An unpaired *t-*test was employed for data comparison between two groups, while one-way ANOVA with Tukey's post hoc test was used for multigroup data comparison.

We first screened the expression of HDAC I (HDAC1, 2, 3, and 8), II (HDAC4, 5, 6, 7, 9, and 10), and IV (HDAC11) in CCA through TCGA database. The results showed higher expression of HDAC3, HDAC7, HDAC10, and HDAC11 in CCA samples compared with the normal samples. Among these, the elevated was associated with lower survival rate of patients with CCA (Figure 1(a)).

Moreover, the results of immunohistochemistry displayed that the positive expression of HDAC3 was significantly increased in CCA tissues compared with normal bile duct tissues (Figure 1(b)). Western blotting data further exhibited higher expression of HDAC3 in cancer tissues than in normal bile duct tissues (Figure 1(c)). The above results indicate that HDAC3 is abundantly expressed in CCA tissues and is related to poor prognosis of patients.

### 3.2. CDM Inhibits the Proliferation, Migration, and Invasion but Promotes Apoptosis and Autophagy of CCA Cells by Inhibiting HDAC3

**Figure 2:**
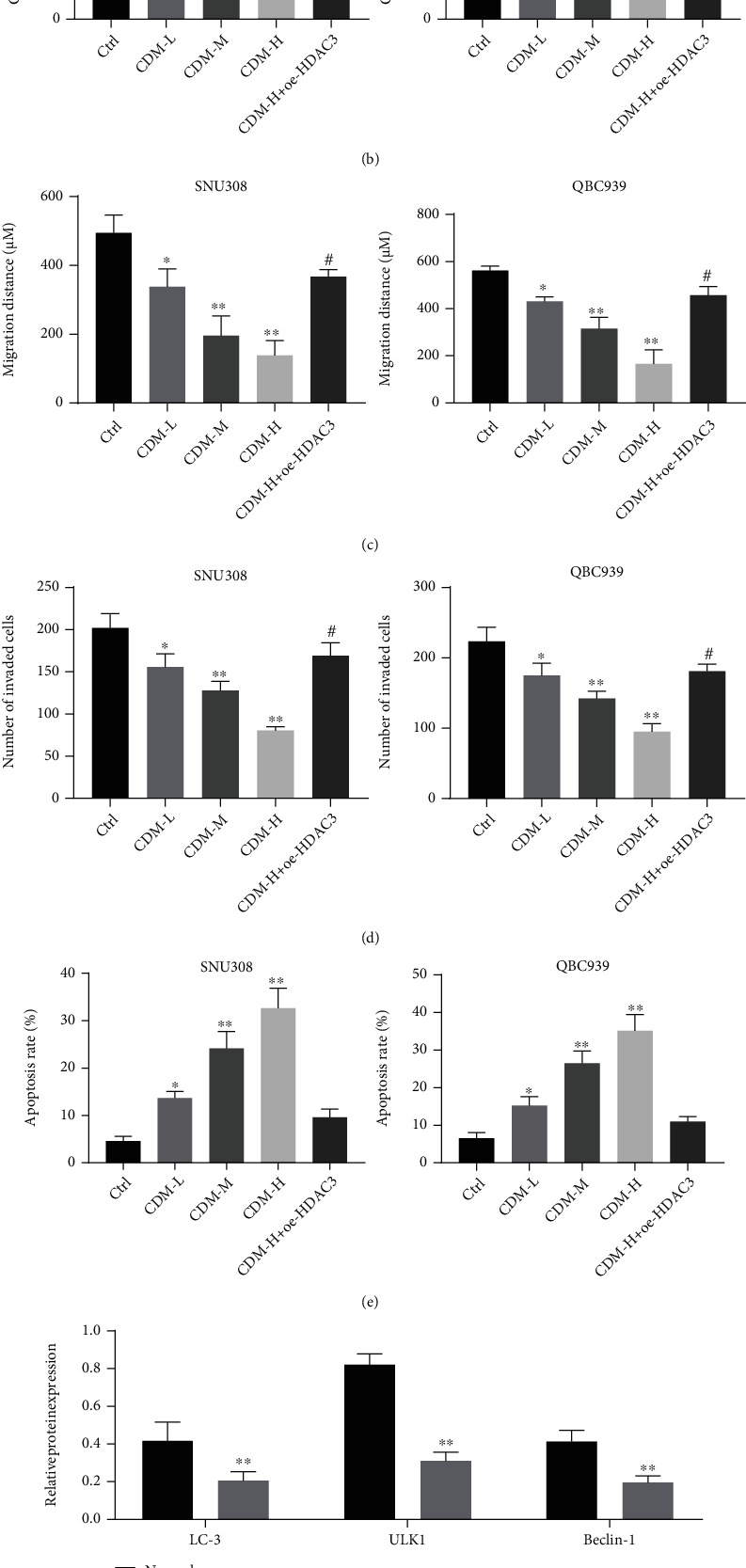
CDM suppresses the proliferation, migration and invasion of CCA cells while stimulating their apoptosis and autophagy by inhibiting HDAC3. (a) Proliferation of QBC939 and SNU308 cells treated with CDM at varied concentrations measured by CCK-8. (b) Colony formation of QBC939 and SNU308 cells treated with CDM-H or combined with oe-HDAC3 measured by colony formation assay. (c) Migration of QBC939 and SNU308 cells treated with CDM-H or combined with oe-HDAC3. (d) Invasion of QBC939 and SNU308 cells treated with CDM-H or combined with oe-HDAC3. (e) Flow cytometric analysis of the apoptosis of QBC939 and SNU308 cells treated with CDM-H or combined with oe-HDAC3. (f) Western blotting of autophagy-related proteins LC-3, ULK1, and Beclin-1 in cancer tissues (*n* = 26) and normal bile duct tissues (*n* = 26). (g) Western blotting of autophagy-related proteins LC-3, ULK1, and Beclin-1 in QBC939 and SNU308 cells treated with CDM-H or combined with oe-HDAC3 or CQ. Data were shown as the mean ± standard deviation. One-way ANOVA with Tukey's post hoc test was used for multigroup data comparison. The cell experiment was repeated three times. ^∗^*p* < 0.05 and ^∗∗^*p* < 0.01, compared with control cells or normal bile duct tissues. ^#^*p* < 0.05, compared with CDM-H-treated cells.

CCK-8 test results showed a gradual decrease in the proliferation of QBC939 and SNU308 cells with increasing CDM concentration (0.1, 0.5, 1.0, 2.5, 5.0, 10.0, and 20.0 *μ*M) (Figure 2(a)). IC50 values of QBC939 and SNU308 were calculated to be 0.4392 *μ*M and 0.3655 *μ*M, respectively. As shown in Figures 2(b)–2(d), colony formation, migration, and invasion of QBC939 and SNU308 cells were reduced in response to treatment with CDM in a dose-dependent manner. Combined treatment with CDM-H and oe-HDAC3 led to higher cell colony formation, migration, and invasion than seen with CDM-H treatment alone. Besides, CDM treatment promoted the apoptosis rate of QBC939 and SNU308 cells, which displayed a dose-dependent response. However, the apoptosis rate of QBC939 and SNU308 cells was augmented following combined treatment with CDM-H and oe-HDAC3 relative to CDM-H treatment alone (Figure 2(e)).

The results of Western blotting suggested that the expression of autophagy-related proteins LC-3, ULK1, and Beclin-1 was decreased in cancer tissues (Figure 2(f)), while CDM treatment led to a dose-dependent increase in their expression. However, the expression of LC-3, ULK1 and Beclin-1 was reduced in QBC939 and SNU308 cells treated with CDM-H+oe-HDAC3 CDM-H+chloroquine (CQ; the autophagy inhibitor) (Figure 2(g)). Taken together, these data confirm that CDM can repress the proliferation, migration, and invasion of CCA cells while promoting their apoptosis and autophagy by inhibiting HDAC3.

### 3.3. CDM Induces Cell Autophagy by Increasing FOXO1 Acetylation Level

**Figure 3:**
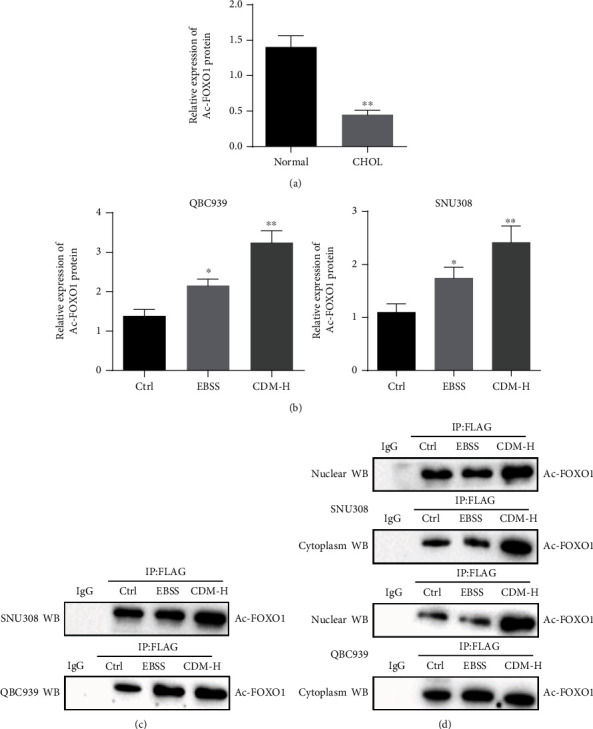
CDM induces cell autophagy by increasing FOXO1 acetylation level. (a) Acetylation level of FOXO1 in cancer tissues (*n* = 26) and normal bile duct tissues (*n* = 26) determined by Western blotting. ^∗^*p* < 0.05 and ^∗∗^*p* < 0.01, compared with normal bile duct tissues. (b) Acetylation level of FOXO1 in QBC939 and SNU308 cells treated with EBSS and CDM-H detected by Western blotting. ^∗^*p* < 0.05 and ^∗∗^*p* < 0.01, compared with control cells. (c) IP analysis of cells, FLAG precipitation and Ac-FOXO1 immunoblotting. (d) IP detection of QBC939 and SNU308 cells, FLAG precipitation and Ac-FOXO1 nuclear and cytosolic immunoblotting. Data were shown as the mean ± standard deviation. One-way ANOVA or repeated measures ANOVA with Tukey's post hoc test was used for multi-group data comparison. The experiment was repeated three times.

Previous studies have reported that FOXO1 acetylation is involved in cell autophagy [18, 21]. Therefore, we speculated that CDM-induced autophagy in CCA cells is related to FOXO1 acetylation. To test this prediction, we first used Western blotting to detect the FOXO1 acetylation level in cancer tissues. The results revealed a lower acetylation level of FOXO1 in cancer tissues than in normal bile duct tissues (Figure 3(a)), demonstrating that CDM-induced autophagy was indeed correlated to FOXO1 acetylation.

Subsequently, we starved cells to induce autophagy. Compared with the control cells, the acetylation level of FOXO1 was higher in the EBSS (starvation treatment) cells (Figure 3(b)). The results of IP also revealed an increase in the acetylation level of FOXO1 in EBSS- and CDM-H-treated cells relative to the control cells (Figure 3(c)). In addition, IP results presented higher FOXO1 acetylation level in the cytoplasm and nucleus of EBSS cells than in control cells (Figure 3(d)). These data demonstrate that CDM can induce cell autophagy by increasing FOXO1 acetylation level.

### 3.4. CDM Promotes FOXO1 Acetylation by Inhibiting HDAC3

**Figure 4:**
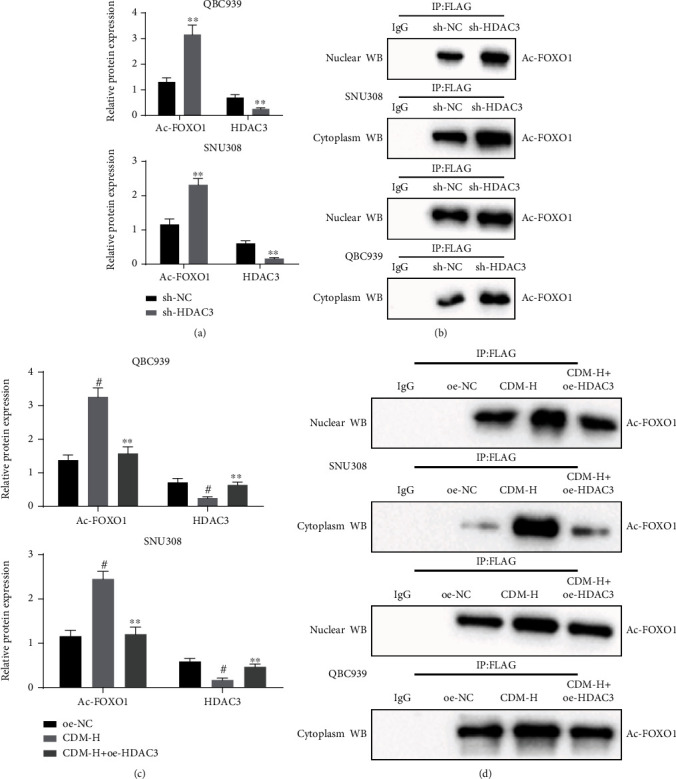
CDM enhances FOXO1 acetylation by inhibiting HDAC3. (a) HDAC3 protein expression and FOXO1 acetylation level in QBC939 and SNU308 cells treated with sh-HDAC3 detected by Western blotting. ^∗^*p* < 0.05 and ^∗∗^*p* < 0.01, compared with cells treated with sh-NC. (b) After inhibiting the expression of HDAC3 in QBC939 and SNU308 cells, IP was performed by FLAG precipitation and nuclear and cytosolic Ac-FOXO1. (c) HDAC3 protein expression and FOXO1 acetylation level in QBC939 and SNU308 cells treated with CDM-H or combined with oe-HDAC3 detected by Western blotting. ^∗∗^*p* < 0.01, compared with cells treated with CDM-H. ^#^*p* < 0.05, compared with cells treated with oe-NC. (d) After overexpressing HDAC3 in QBC939 and SNU308 cells, IP was performed by FLAG precipitation and nuclear and cytosolic Ac-FOXO1. Data were shown as the mean ± standard deviation. An unpaired *t-*test was employed for data comparison between two groups, while one-way ANOVA with Tukey's post hoc test was used for multi-group data comparison. The experiment was repeated three times.

It has been reported that HDAC3 activates the transcription of FOXO1 [22]. We proceeded in this study to investigate whether CDM induced FOXO1 acetylation by inhibiting HDAC3. First, QBC939 and SNU308 cells were treated with sh-HDAC3. The results of Western blotting revealed that, compared with the sh-NC treatment, HDAC3 protein expression was decreased, while the FOXO1 acetylation level was enhanced in the sh-HDAC3-treated cells (Figure 4(a)). Furthermore, after nuclear and cytosolic separation of the cells, the FOXO1 acetylation level in the nucleus and cytoplasm was detected by IP. Here the results showed that the FOXO1 acetylation level was also increased in the nucleus and cytoplasm of cells treated with sh-HDAC3 (Figure 4(b)), thus consistently showing that inhibiting HDAC3 could accelerate the acetylation of FOXO1.

Subsequently, QBC939 and SNU308 cells were transfected with plasmids overexpressing HDAC3 and then treated with CDM. As illustrated in Figure 4(c), the protein expression of HDAC3 was reduced while FOXO1 acetylation level was decreased in cells treated with CDM-H. The effects of CDM-H on HDAC3 protein expression and FOXO1 acetylation level were negated by further overexpression of HDAC3. After nuclear and cytosolic separation of the cells, Ac-lysine was collected to detect the FOXO1 acetylation level. IP results shown in Figure 4(d) indicated that the FOXO1 acetylation level was greater in the nucleus and cytoplasm of CDM-H-treated cells than in the oe-NC-treated cells, while opposite results were noted in the presence of CDM-H+oe-HDAC3. These results serve as new evidence that inhibition of HDAC3 may be responsible for the promotion of FOXO1 acetylation by CDM.

### 3.5. CDM Arrests the Growth of CCA by Regulating the HDAC3/FOXO1 Axis In Vivo

**Figure 5:**
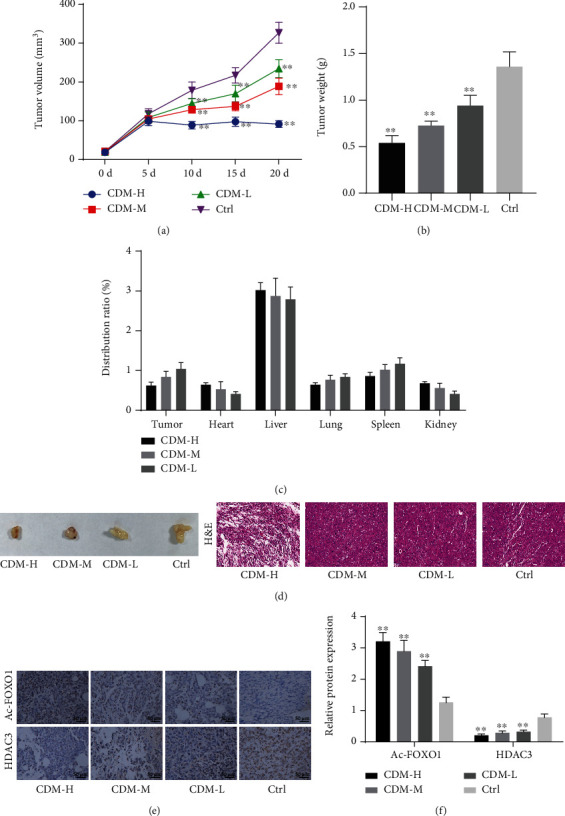
CDM suppresses the growth of CCA *in vivo*. (a) Tumor volume of control and CDM mice. (b) Tumor weight of control and CDM mice. (c) The distribution of CDM in the tumor and major organs of nude mice. (d) Representative images showing xenografts in nude mice and H&E staining of tumor tissues of control and CDM mice. (e) Immunohistochemical staining of HDAC3 protein and FOXO1 acetylation level in tumor tissues of control and CDM mice. (f) Western blotting of HDAC3 protein and FOXO1 acetylation level in tumor tissues of control and CDM mice. ^∗^*p* < 0.05 and^∗∗^*p* < 0.01, compared with control mice. Data were shown as the mean ± standard deviation. One-way ANOVA with Tukey's post hoc test was used for multigroup data comparison and repeated measures ANOVA with Tukey's post hoc test was applied to compare data at different time points. *n* = 6 for mice in each group.

The aforementioned results verified that CDM induced cell autophagy by inhibiting HDAC3 and inducing FOXO1 acetylation in QBC939 and SNU308 cells. Next, we aimed to further investigate whether CDM played an antitumor role in nude mice bearing a CCA xenograft. The mouse model of CCA was established through injection of human CCA QBC939 cells. Three dose groups were set, including CDM-L (22.5 mg/kg), CDM-M (45 mg/kg), and CDM-H (90 mg/kg). As shown in Figures 5(a) and 5(b) and Supplementary Table 1, CDM inhibited the growth of CCA in a dose-dependent manner. In addition, further analysis of the distribution of CDM in tumors and major organs showed that CDM was mainly enriched in the liver of the nude mice (Figure 5(c)), indicating that CDM was mainly metabolized by the liver. Besides, H&E staining results illustrated that tumor cells in the tumor tissues of control mice were tightly arranged, with large nuclei, less cytoplasm, and obvious cell atypia. Following treatment with CDM, the cells were relatively reduced in number and replaced by fibrous tissues. More fibrous tissues were present with increasing CDM dose (Figure 5(d)). At the same time, the results of immunohistochemical staining and Western blotting suggested a decline in the HDAC3 expression in the tumor tissues of CDM-treated mice, while FOXO1 acetylation level was increased in a dose-dependent manner (Figures 5(e) and 5(f)). The above results indicate that CDM can inhibit the growth of CCA *in vivo*.

**Figure 6:**
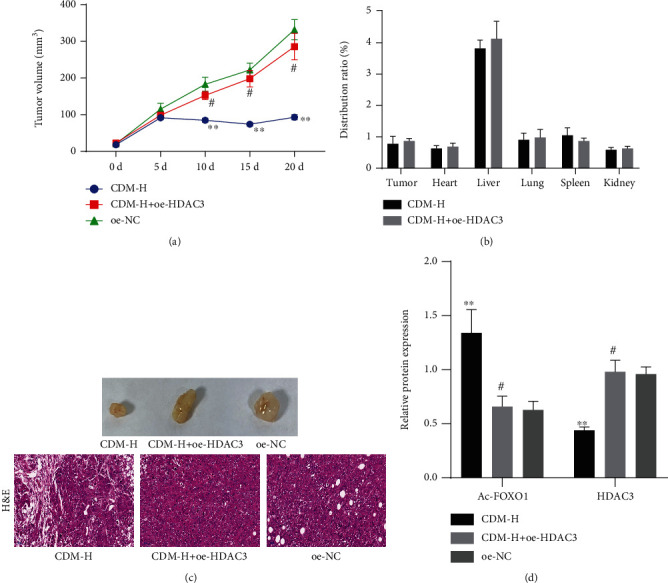
CDM retards the growth of CCA in nude mice by regulating the HDAC3/FOXO1 axis. (a) Tumor volume of mice treated with CDM-H or combined with oe-HDAC3. (b) The distribution of CDM in the tumor and major organs of mice treated with CDM-H or combined with oe-HDAC3. (c) Representative images showing xenografts in nude mice and H&E staining of tumor tissues of mice treated with CDM-H or combined with oe-HDAC3. (d) Western blotting of HDAC3 protein and FOXO1 acetylation level in tumor tissues of mice treated with CDM-H or combined with oe-HDAC3. ^∗∗^*p* < 0.01, compared with oe-NC-treated mice. ^#^*p* < 0.05, compared with CDM-H-treated mice. Data are shown as the mean ± standard deviation. One-way ANOVA with Tukey's post hoc test was used for multigroup data comparison and repeated measures ANOVA with Tukey's post hoc test was applied to compare data at different time points. *n* = 6 for mice in each group.

Finally, we sought to investigate whether the anti-tumor effect of CDM *in vivo* was correlated with the regulation of the HDAC3/FOXO1 axis. As shown in Figure 6(a), CDM-H treatment delayed the growth of tumors while further HDAC3 overexpression accelerated the tumor growth. Besides, CDM was still mainly distributed in the liver of mice treated with CDM and oe-HDAC3 (Figure 6(b)). As shown in Figure 6(c), tumor cells in the tumor tissues of oe-NC-treated mice were tightly arranged, with large nuclei, less cytoplasm, and obvious cell atypia. Following treatment with CDM-H, the cells were largely reduced and replaced by fibrous tissues. Conversely, treatment with CDM-H+oe-HDAC3 increased the number of tumor cells and reduced fibrous tissues formation. In addition, the results of Western blotting suggested a decline in HDAC3 expression in the tumor tissues of CDM-H-treated mice, while FOXO1 acetylation level was increased. Further overexpression of HDAC3 led to opposite results (Figure 6(d)). Overall, these lines of evidence demonstrate that CDM could inhibit HDAC3 expression and increase FOXO1 acetylation, thus preventing the growth of CCA *in vivo*.

## 4. Discussion

CCA is a deadly disease, such that surgery and adjunct treatments are curative in only a few cases [23]. The search to improve the efficacy of CCA treatments calls upon obtaining a better understanding of its molecular pathogenesis of CCA to support the development of rational therapies. In this paper, we studied the antitumor effects and underlying mechanism of CDM in CCA cells. Our experimental results showed that CDM elicited anti-tumor effects by inhibiting HDAC3-mediated deacetylation of FOXO1.

According to previous reports, CDM inhibits the proliferation of glioma cells, lung cancer, pancreatic cancer, and myeloma cells [24–26]. In Jurkat and HUT-78 cells, treatment with CDM (2 *μ*M) leads to downregulation of HDAC3 expression, thus inducing necroptosis [27]. Our present research obtained similar results in CCA cells, while our cell migration and colony formation experiments suggested that CDM inhibited the proliferation of CCA cells in a dose-dependent manner, which also promoted cell autophagy. Previous research has indicated that miR-373 inhibits autophagy of CCA cells by targeting ULK1 and promoting apoptosis [28]. Dihydroartemisinin, which was found to have antitumor activity in a variety of human cancers, induces cell apoptosis and autophagy-dependent cell death of CCA cells *via* the DAPK1-BECLIN1 pathway [29]. Using autophagy as an adaptive mechanism, cancer cells can survive under conditions of extreme stress in the tumor microenvironment and can also promote the invasiveness and resistance of anti-cancer drugs. In the initial stage of CCA development, cell autophagy can be impaired, and the associated accumulation of LC3-II and p62 may reflect defects in the late processing of autophagosomes, rather than an increase in autophagy rate. In preclinical studies, autophagy modulators can promote CCA cell death, reduce invasion, and render CCA cells sensitive to chemotherapy. Inhibition of autophagy may promote oncogenic transformation of bile duct cells, and impaired autophagy caused by inactivation of Beclin1 may promote the malignant phenotype of CCA [30]. Inhibition of autophagy has thus been considered to be a new strategy to prevent the growth of cancer cells. Results of the mechanistic investigation in the current study suggest that CDM exerts an antitumor effect by inhibiting HDAC3 to promote cell autophagy, based on findings of reduced expression of the autophagy-related proteins LC-3, Ulk1, and Beclin1. CDM has been reported to be an HDAC inhibitor [31], which in combination with rituximab can inhibit the growth of diffuse large B-cell lymphoma tumors [15]. Besides, inhibition of HDAC3 expression can decrease the proliferation of myeloma and CCA cells [32, 33]. Our present results suggest that HDAC3 has a clear correlation with clinical CCA and is involved in the associated regulation of cell autophagy.

Furthermore, the current results in CCA cells showed that CDM treatment increased FOXO1 acetylation level, which led to the inhibition of HDAC3 activity and anti-CCA effects. As a potential therapeutic target, FOXO1 can regulate the autophagy flux of human CCA cells [17]. Moreover, FOXO1 acetylation is closely associated with cell autophagy [18, 21]. It is furthermore reported that FOXO1 exerts its anti-tumor action by regulating the autophagy of tumor cells [17]. Thus, FOXO1 deacetylation promotes cell autophagy [34], but inhibiting the autophagy level of cancer cells can enhance their chemotherapeutic sensitivity [35–37]. To conclude, our present findings elaborate that CDM ameliorates CCA by inhibiting the expression of HDAC3 and further promoting FOXO1 acetylation, which activates the autophagy of CCA cells.

## 5. Conclusions

**Figure 7:**
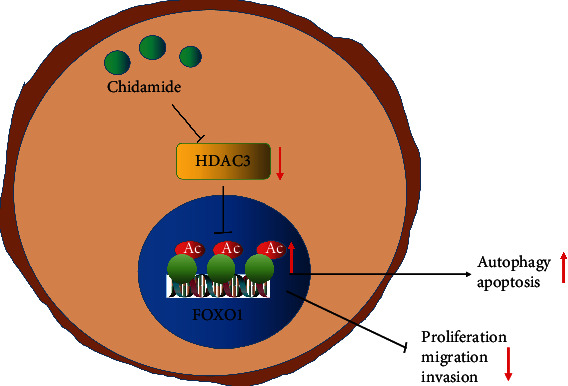
Schematic diagram of the mechanism by which CDM affects CCA. CDM inhibits expression of HDAC3 and thus promotes FOXO1 acetylation level, thereby inhibiting the proliferation, migration, and invasion of CCA cells, promoting cell apoptosis and autophagy, and ultimately arresting the growth of CCA.

This study demonstrates that CDM has a significant tumor suppressing effect on CCA and may eventually provide a new treatment approach for CCA. CDM suppresses HDAC3 expression and induces acetylation of FOXO1, thus impeding the proliferation, migration, and invasion of CCA cells, while promoting cell apoptosis and autophagy, ultimately arresting the growth of CCA (Figure 7). We propose that CDM may act synergistically with other chemotherapy drugs in the treatment of CCA. As for FOXO1, further investigations may reveal how FOXO1 could improve the drug sensitivity of CCA tumors and discourage their otherwise inexorable progression.

## Data Availability

The datasets generated/analyzed during the current study are available.

## Supplementary Materials

Supplementary Materials Supplementary Table 1: the effect of CDM on the tumor volume and weight of QBC939 tumor-bearing nude mice. ^a^TIR: tumor inhibition rate. ^b^Data were shown as the mean ± standard deviation. *n* = 6 for mice in each group. ^∗^*p* < 0.05; ^∗∗^*p* < 0.01 compared with control mice.
