# Supplementary material for: Chidamide Suppresses the Growth of Cholangiocarcinoma by Inhibiting HDAC3 and Promoting FOXO1 Acetylation
Source: Stem Cells Int. 2022 Jan 28;2022:3632549. doi: 10.1155/2022/3632549 (PMC8816583; doi:10.1155/2022/3632549)
Supplement: Supplementary Materials — Supplementary Table 1: the effect of CDM on the tumor volume and weight of QBC939 tumor-bearing nude mice. aTIR: tumor inhibition rate. bData were shown as the mean ± standard deviation. n = 6 for mice in each group. ∗p < 0.05; ∗∗p < 0.01 compared with control mice. [file 3632549.f1.doc]

**Supplementary Table 1 The effect of CDM on the tumor volume and weight of QBC939 tumor-bearing nude mice.**

| Group | Dose | TV (mm^3^) | | Tumor weight (g) | TIR (%)^a^ |
| --- | --- | --- | --- | --- | --- |
|  |  | day 0 | day 15 |  |  |
| CDM-H | 90.00 mg/kg | 18.69 ± 2.75^b^ | 97.51 ± 11.80** | 0.54 ± 0.08** | 63.49 ± 7.85 ** |
| CDM-M | 45.00 mg/kg | 20.81 ± 1.93 | 137.12 ± 11.43** | 0.73± 0.05** | 46.19 ± 5.29** |
| CDM-L | 22.50 mg/kg | 19.15 ± 3.01 | 169.75 ± 21.36** | 0.94 ± 0.11** | 17.82 ± 2.53** |
| Control | 0 | 20.86 ± 4.12 | 216.95 ± 19.68 | 1.36 ± 0.16 | 1.83 ± 0.29 |

^a^ TIR: tumor inhibition rate

^b^ Data were shown as mean ± standard deviation. n = 6 for mice in each group. **p* < 0.05; ***p* < 0.01 compared with control mice.
